# Multimodal noninvasive and invasive imaging of extracranial venous abnormalities indicative of CCSVI: Results of the PREMiSe pilot study

**DOI:** 10.1186/1471-2377-13-151

**Published:** 2013-10-20

**Authors:** Robert Zivadinov, Yuval Karmon, Kresimir Dolic, Jesper Hagemeier, Karen Marr, Vesela Valnarov, Cheryl L Kennedy, David Hojnacki, Ellen M Carl, L Nelson Hopkins, Elad I Levy, Bianca Weinstock-Guttman, Adnan H Siddiqui

**Affiliations:** 1 Buffalo Neuroimaging Analysis Center, Department of Neurology, University at Buffalo, State University of New York, Buffalo, NY, USA; 2 Department of Neurology, The Jacobs Neurological Institute, University at Buffalo, State University of New York, Buffalo, NY, USA; 3 Departments of Neurosurgery and Radiology, University at Buffalo, State University of New York, Buffalo, NY, USA; 4 Department of Neurology, School of Medicine and Biomedical Sciences, Buffalo Neuroimaging Analysis Center, 100 High St., Buffalo, NY 14203, USA

**Keywords:** Chronic cerebrospinal venous insufficiency (CCSVI), Multiple sclerosis, Multimodal imaging, Doppler sonography, Magnetic resonance venography, Catheter venography, Intravascular ultrasound, Extracranial venous anomalies

## Abstract

**Background:**

There is no established noninvasive or invasive diagnostic imaging modality at present that can serve as a ‘gold standard’ or “benchmark” for the detection of the venous anomalies, indicative of chronic cerebrospinal venous insufficiency (CCSVI). We investigated the sensitivity and specificity of 2 invasive vs. 2 noninvasive imaging techniques for the detection of extracranial venous anomalies in the internal jugular veins (IJVs) and azygos vein/vertebral veins (VVs) in patients with multiple sclerosis (MS).

**Methods:**

The data for this multimodal imaging comparison pilot study was collected in phase 2 of the “Prospective Randomized Endovascular therapy in Multiple Sclerosis” (PREMiSe) study using standardized imaging techniques. Thirty MS subjects were screened initially with Doppler sonography (DS), out of which 10 did not fulfill noninvasive screening procedure requirements on DS that consisted of ≥2 venous hemodynamic extracranial criteria. Accordingly, 20 MS patients with relapsing MS were enrolled into the multimodal diagnostic imaging study. For magnetic resonance venography (MRV), IJVs abnormal findings were considered absent or pinpoint flow, whereas abnormal VVs flow was classified as absent. Abnormalities of the VVs were determined only using non-invasive testing. Catheter venography (CV) was considered abnormal when ≥50% lumen restriction was detected, while intravascular ultrasound (IVUS) was considered abnormal when ≥50% restriction of the lumen or intra-luminal defects or reduced pulsatility was found. Non-invasive and invasive imaging modality comparisons between left, right and total IJVs and between the VVs and azygos vein were performed. Because there is no reliable way of non-invasively assessing the azygos vein, the VVs abnormalities detected by the non-invasive testing were compared to the azygos abnormalities detected by the invasive testing. All image modalities were analyzed in a blinded manner by more than one viewer, upon which consensus was reached. The sensitivity and specificity were calculated using contingency tables denoting the presence or absence of vein-specific abnormality findings between all imaging modalities used individually as the benchmark.

**Results:**

The sensitivity of CV + IVUS was 68.4% for the right and 90% for the left IJV and 85.7% for the azygos vein/VVs, compared to venous anomalies detected on DS. Compared to the venous anomalies detected on MRV, the sensitivity of CV + IVUS was 71.4% in right and 100% in left IJVs and 100% in the azygos vein/VVs; however, the specificity was 38.5%, 38.9% and 11.8%, respectively. The sensitivity between the two invasive imaging techniques, used as benchmarks, ranged from 72.7% for the right IJV to 90% for the azygos vein but the IVUS showed a higher rate of venous anomalies than the CV. There was excellent correspondence between identifying collateral veins on MRV and CV.

**Conclusions:**

Noninvasive DS screening for the detection of venous anomalies indicative of CCSVI may be a reliable approach for identifying patients eligible for further multimodal invasive imaging testing of the IJVs. However, the noninvasive screening methods were inadequate to depict the total amount of azygos vein/VVs anomalies identified with invasive testing. This pilot study, with limited sample size, shows that both a non-invasive and invasive multimodal imaging diagnostic approach should be recommended to depict a range of extracranial venous anomalies indicative of CCSVI. However, lack of invasive testing on the study subjects whose results were negative on the DS screening and of healthy controls, limits further generalizibility of our findings. In addition, the findings from the 2 invasive techniques confirmed the existence of severe extracranial venous anomalies that significantly impaired normal blood outflow from the brain in this group of MS patients.

## Background

In 2009, Zamboni et al. described a vascular condition in patients with multiple sclerosis (MS) named chronic cerebrospinal venous insufficiency (CCSVI), as a restriction of main extracranial cerebrospinal venous routes that is caused by anomalies, which interfere with normal intra-cranial venous outflow [1,2].

CCSVI implies a pathological condition for which noninvasive diagnosis is based mainly on the detection of ≥2 positive venous hemodynamic (VH) criteria on color Doppler sonography (DS) in the extracranial (neck) and intra-cranial veins by assessing 5 proposed VH criteria [1,2]. The non-invasive DS diagnosis of CCSVI has been originally confirmed by the use of invasive catheter venography (CV) examination [1,3]. On CV, extracranial pathology was considered significant if the stenosis detected by venous diameter reduction was equal to or exceeded 50% in any of the internal jugular veins (IJVs) or the azygos vein [1]. Because of the advantages of the DS being noninvasive and providing high-resolution images with real time dynamic information of functional and structural venous anomalies at a relatively low cost, it has been promoted as a method of choice for the screening of CCSVI [4].

However, subsequent studies showed that the reproducibility of DS VH criteria depends on the training and skills of the operator and that they are not easily blinded or standardized in either research or clinical settings [5-8]. Moreover, the pathologic value of the CCSVI diagnosis by DS is controversial because the categorical construct of the CCSVI diagnosis (≥2 and <2 VH DS criteria fulfilled) most likely contributed to explaining major inconsistencies in the prevalence of findings of CCSVI between different studies in MS ranging from 0-100% [1,5,9-17]. In addition, CCSVI is not specific for MS, as it was described in a substantial number of healthy controls and patients with other neurological diseases [5,10,18].

At this time, there is no established noninvasive or invasive diagnostic imaging modality that can serve as a “gold standard/benchmark” for the detection of extracranial venous anomalies, indicative of CCSVI. Each proposed imaging modality for the screening and diagnosis of CCSVI has its own advantages and disadvantages [19]. Therefore, most likely, only a multimodal imaging approach will represent the most comprehensive means for the screening, diagnosis as well as monitoring for these extracranial venous anomalies.

Against this background,the goal of this study was to define and reliably detect extracranial venous anomalies, indicative of CCSVI in the IJVs and azygos vein/vertebral veins (VVs) of patients with MS. We investigated the sensitivity and specificity of 2 invasive vs. 2 noninvasive imaging techniques for the detection of these extracranial venous anomalies.

## Methods

### Study design

Prospective Randomized Endovascular Therapy in Multiple Sclerosis (PREMiSe; ClinicalTrials.gov. NCT01450072) is an endovascular angioplasty pilot study, planned in two phases, which included 30 patients with MS. Phase I was an open-label safety phase that included 10 patients with CCSVI, whereas phase 2 was sham-controlled, randomized, double-blind and included 20 patients. Patients obtained their baseline diagnostic assessment between June of 2010 and March of 2012. Phase 1 was planned to optimize work flow, standardize protocols and blinding methodologies for Phase 2 with the use of invasive imaging techniques (CV and IVUS) for a more accurate extracranial venous drainage assessment and venous angioplasty; hence, the imaging protocols were not performed in a standardized fashion. Therefore, the present study describes baseline diagnostic multimodal standardized imaging assessments limited to patients included in Phase 2. The study was approved by the Institutional Review Board of the University at Buffalo and was overseen by an independent data-safety monitoring committee. All participants gave their written informed consent.

Inclusion criteria for phase 2 of the PREMiSe study at screening were as follows: 1) age 18–65 years; 2) active-relapsing MS defined as having one relapse within the past 12 months; or 3) presence of gadolinium-enhancing lesion(s) on post-contrast MRI within the previous 3 months; 4) being on concomitant disease-modifying treatments excluding natalizumab; 5) having relapsing MS, [20] Expanded Disability Status Scale (EDSS) [21] of 0–5.5; and 6) fulfilling ≥2 CCSVI VH extracranial DS criteria [1].

Exclusion criteria for the PREMiSe study at screening were: an acute relapse, disease progression and/or steroid treatment within 30 days preceding study entry, pre-existing medical conditions known to be associated with brain pathology (e.g., neurodegenerative disorder, cerebrovascular disease, positive history of alcohol abuse), severe peripheral chronic venous insufficiency, severe contrast media allergy (anaphylaxis) and abnormal renal function.

### Doppler sonography

**Figure 1:**
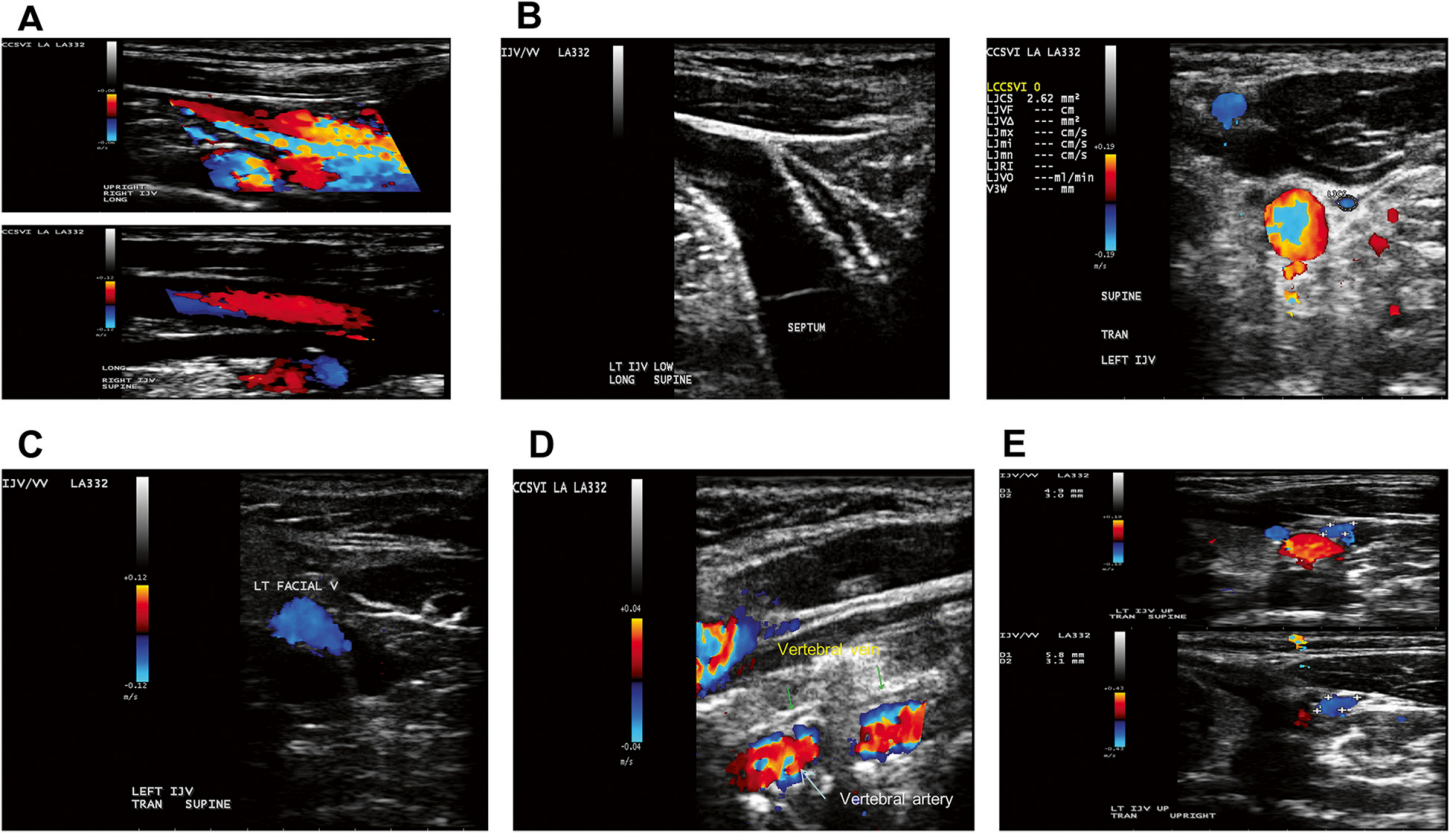
**Doppler sonography venous hemodynamic (VH) criteria for diagnosis of chronic cerebrospinal venous insufficiency (CCSVI). A.** VH criteria 1 - Reflux or bidirectional flow noted in the internal jugular vein (IJV) with the head in supine and upright position, **B.** VH criteria 3 - B-mode anomaly and/or stenosis: septum noted in the lower IJV and stenosis noted in the left IJV with a cross sectional area (CSA) ≤0.3 cm^2^; **C.** VH criteria 4 - No flow detected in the IJV until the facial vein enters IJV, IJV demonstrates no flow above facial vein entry; **D.** VH criteria 4 - No flow in vertebral veins (VV) despite low wall filter and low velocity scale; **E.** VH criteria 5 - Negative delta CSA – IJV cross sectional area is larger in the upright position than in the supine position resulting in a negative delta CSA (supine IJV CSA 11.54 – upright IJV CSA 12.90 = −1.36 mm).

Patients needed to fulfill noninvasive DS extracranial VH criteria, [1] to qualify for the invasive diagnostic part of the study. DS examination was performed using a color-coded scanner (MyLab Gold 25; Esaote-Biosound, Irvine, California) equipped with a 7 to 10-Mhz transducer to examine IJVs and vertebral veins (VVs) morphology as well as hemodynamics. The detailed scanning protocol and validation were previously reported [5,6,8]. Briefly, the following 5 VH parameters indicative of CCSVI were investigated (Figure 1): 1) Reflux/bidirectional flow in the IJVs and/or in the VVs in the sitting and supine positions, defined as flow directed towards the brain for a duration of >0.88 s; 2) Reflux/bidirectional flow in the deep cerebral veins defined as “reverse flow” for a duration of 0.5 s in one of the intra-cranial veins; 3) B-mode abnormalities or stenosis in IJVs. IJV stenosis was defined as a cross-sectional area (CSA) ≤0.3 cm^2^; 4) Flow that is not Doppler-detectable in IJVs and/or VVs despite multiple deep breaths and 5) Reverted postural control of the main cerebral venous outflow pathway by measuring the difference in the CSA in the IJVs in the supine and upright positions. A subject was considered positive at screening in phase 2 if ≥2 VH extracranial criteria (1, 3, 4, and 5) were fulfilled.

### Magnetic resonance venography

**Figure 2:**
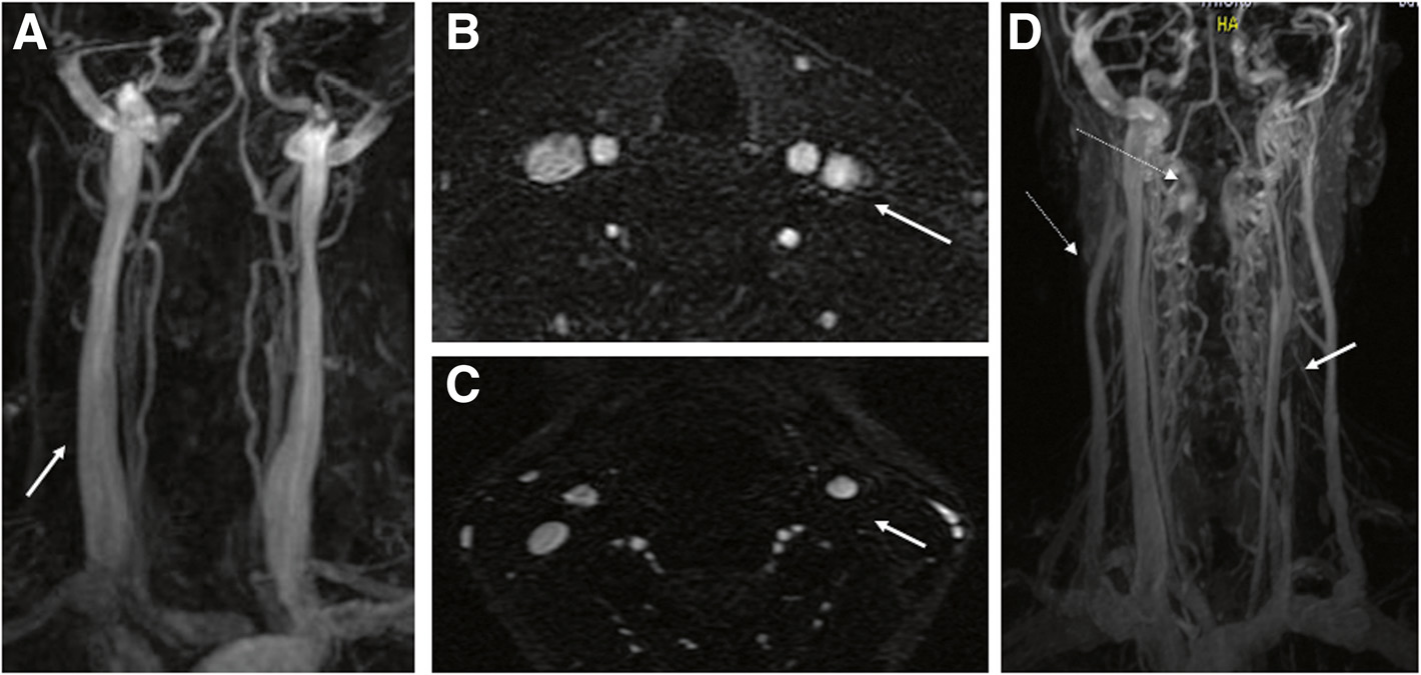
**Example of normal and abnormal flow morphology in internal jugular vein (IJV) on magnetic resonance venography (3D TRICKS and axial 2D TOF).** Normal **(A & B)** flow morphology in both IJV (arrows). Abnormal (absent flow) **(C &D)** flow in the left internal jugular vein (arrow) with prominent collateral veins (dotted arrows).

All subjects were examined on a 3 T GE Signa Excite HD 12.0 scanner (General Electric, Milwaukee, WI). A multi-channel head and neck (HDNV) coil was used to acquire the following sequences: an unenhanced 2D-Time of Flight (TOF) and enhanced 3D-Time Resolved Imaging of Contrast KineticS (TRICKS), as previously described [6,8,22-24]. The parameters used for TOF were: TR/TE 17/4.3 msec (repetition/echo time), flip angle of 70 degrees, 1.5 mm slice thickness, field of view (FOV) = 220 mm, acquisition matrix 320/192, phase FOV 75%, for an in-plane resolution (IPR) of 0.7 mm × 1.1 mm and acquisition in axial scan plane. The parameters used for TRICKS were: TR/TE 4.2/1.6 msec, flip angle of 30 degrees, 2 mm slice thickness, FOV = 340 mm, acquisition matrix 320/192, phase FOV 75%, IPR = 1.1 mm × 1.8 mm and acquisition in coronal scan plane. Intravenous gadolinium contrast (Omniscan®, GE Healthcare, Princeton NJ) was injected at a rate of 2 ml/s using a pressure injector followed by a 20 ml saline flush. The total volume of contrast was 20 ml. After acquisition of a 12 second mask (pre-contrast phase), the scanning of subsequent phases began simultaneously with the intravenous injection. The scan protocol consisted of 18 phases of acquisition, each of a 5 second duration. The flow morphology, indicative of anatomical stenoses, of IJVs was assessed on axial source TOF images, as well as on axial reconstructed TRICKS images, as previously described [22]. The IJV flow was evaluated on an ordinal scale ranging from absent (no visible flow) to ellipsoidal (patent lumen) and defined in 5 qualitative flow categories: absent, pinpoint, flattened, crescentic and ellipsoidal. Only absent or pinpoint flow of the IJVs was considered to be abnormal (Figure 2), while the flow of the VVs was classified as absent/present.

In this study, we also assessed the presence and number of collateral veins, as previously described [22]. These included anterior and external jugular veins, facial veins, thyroid veins as well as deep cervical veins (Figure 2).

### Catheter venography

**Figure 3:**
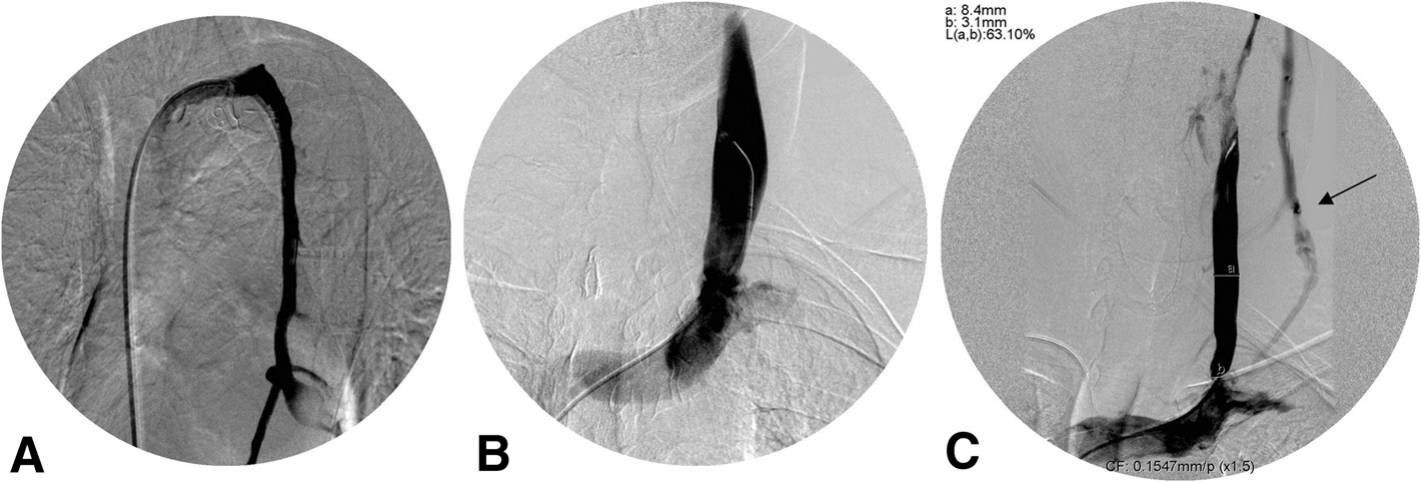
**Catheter venography of azygos and internal jugular veins (IJVs).** Example of normal patent lumen of the azygos vein **(A)** and left internal jugular vein (IJV) **(B)**. Significant stenosis of the distal left IJV **(C)** with prominent collateral vein (arrow).

The detailed protocol of the diagnostic CV was recently reported [25]. Under conscious sedation using local anesthesia, an 8-French sheath was inserted using a modified Seldinger technique into the common femoral vein. Through this sheath, a guide catheter (5-French, 90-cm-long Head Hunter, Terumo Europe, Leuven, Belgium) was advanced through the inferior vena cava, across the right atrium into the superior vena cava. Catheterization proceeded to the azygos vein outlet into the superior vena cava. With the help of a hydrophilic guide wire (0.035-inch diameter Radiofocus Guide Wire M, Terumo Europe), the catheter was advanced inside the azygos vein until it neared the confluence with the hemi-azygos vein at the level of the diaphragm. An autoinjector was used to instill 9 ml of contrast medium (Visipaque, iodixanol, GE HealthCare; 270 mg/mL) at a constant rate of 3 ml/sec. Subtraction digital CV of the azygos vein was completed, with a right posterior oblique projection (range, 15°-25°) and an extended recording time. In this way, it was possible to achieve complete opacification of the system of origin of the azygos vein and hemi-azygos veins up to the ascending lumbar veins. Subsequently, the right IJV and left IJV were approached, in that order. With the help of the guide wire, the catheter was moved inside each IJV up to the junction with the jugular bulb (skull base). Contrast medium (12 ml) was injected at a constant rate of 3 ml/sec. Subtraction digital CV of each IJV was completed with an anterior-posterior projection and an extended recording time. Significant stenosis was considered to be any venous lumen diameter reduction ≥50% (Figure 3).

Collateral draining veins during IJV injection were classified to either epidural plexus or other collaterals (mainly anterior and external jugular veins, not along the spinal cord). Azygos vein collaterals were classified as either epidural (spinal) or other collaterals.

### Intravascular ultrasound

**Figure 4:**
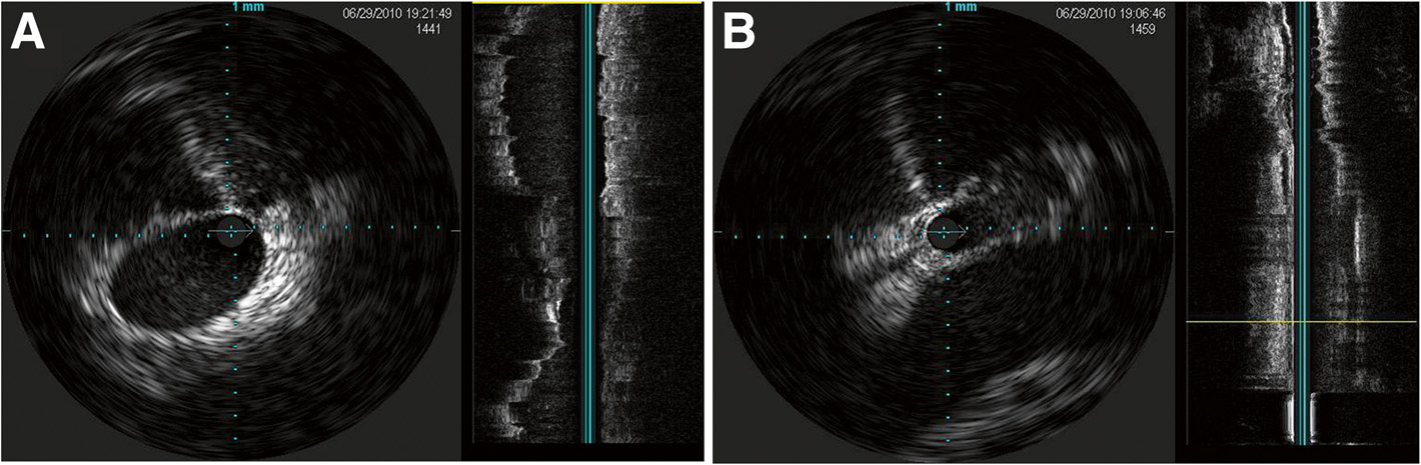
**Example of intravascular ultrasound in the internal jugular vein.** Normal patent lumen **(A)** and stenotic lumen **(B)**.

The detailed protocol of the diagnostic IVUS was recently reported. [25] IVUS (Eagle Eye platinum catheter −20 MHz probe; Volcano s5/s5i Imaging system; Volcano, San-Diego, CA) was consistently performed, independent of the stenosis, to identify venous anomalies. Significant stenosis was considered to be any venous lumen diameter reduction ≥50% (Figure 4) and was calculated as the ratio between the minimal diameter of the vein in any of the axial images and the maximal diameter of the vein in any of the images. All identified stenoses were confirmed to be structural and not physiological by asking the patient to perform a Valsalva maneuver during the IVUS study.

Additional abnormal predefined IVUS abnormalities included the presence of various intra-luminal defects including webs, fibrotic annulus-like constrictions, flaps, septa and vein divided into multiple channels, intra-luminal hyperechoic filling defect (IHFD) and double parallel lumen (DPL). IVUS scans were also interpreted for reduced respiratory pulsatility or normal pulsatility (presence or absence of expansion movements of the vein wall according to respiratory excursions [10-20/min]).

### Multimodal imaging comparisons

**Figure 5:**
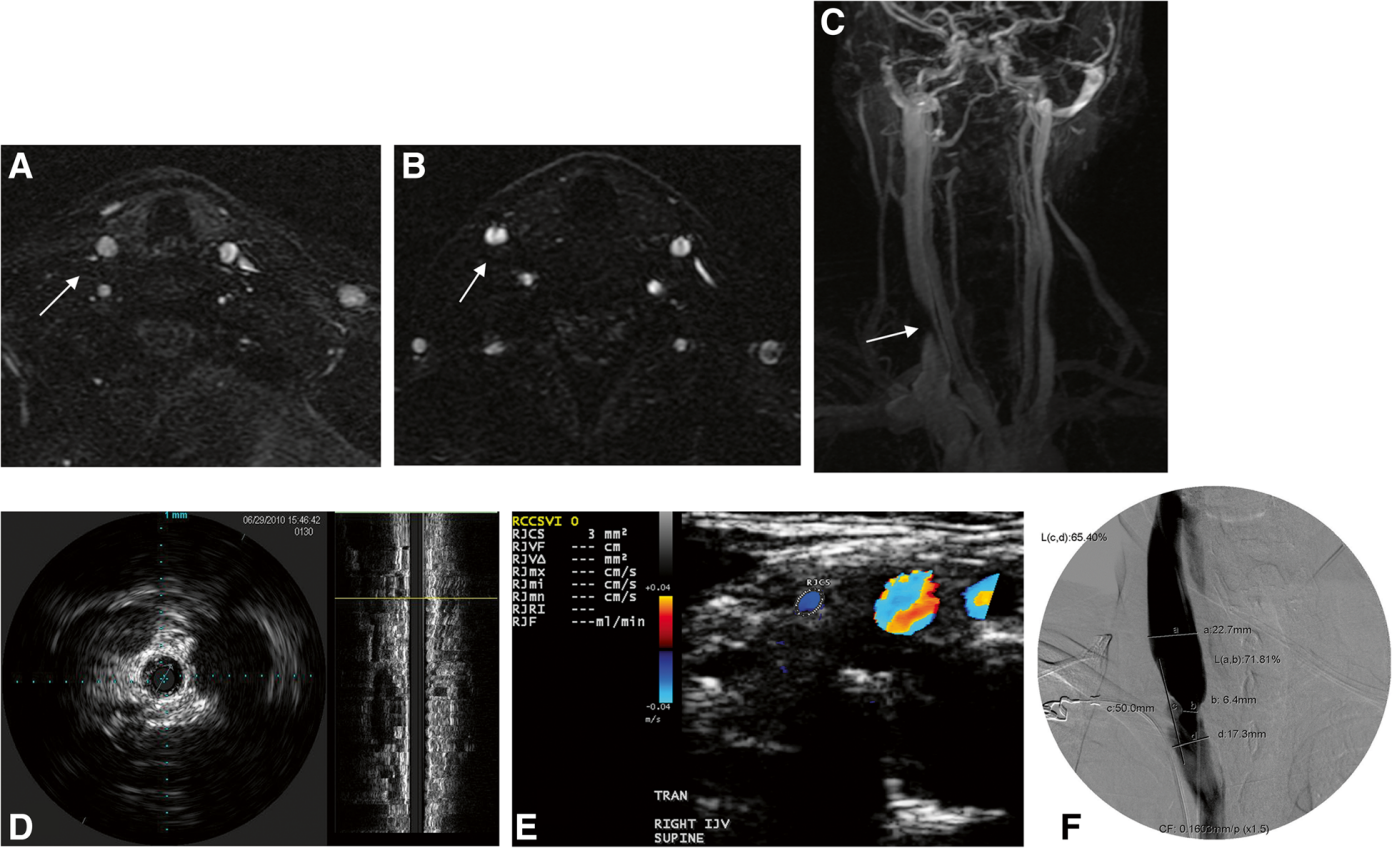
**Multimodal imaging approach of extracranial neck veins.** Axial 2D time-of-flight **(A)**, enhanced 3D-time resolved imaging of contrast kinetics **(B &C)**, intravascular sonography **(D)**, Doppler sonography **(E)**, and catheter venography **(F)** all showing venous abnormality of the right internal jugular vein (significant narrowing-arrows) in the same patient.

Non-invasive and invasive imaging modality comparisons were performed between the left, right and total IJVs and between the VVs and azygos vein. The criteria for the comparison of noninvasive and invasive findings are shown in Table 1. Figure 5 shows a comparison between 2 noninvasive and 2 invasive imaging techniques.

**Table 1:** Multimodal criteria for the detection of abnormal findings in the internal jugular veins and in azygos vein/vertebral veins by using 2 noninvasive and 2 invasive imaging techniques

**Vein territory**	**DS criteria for abnormal findings**	**MRV criteria for abnormal findings**	**CV criteria for abnormal findings**	**IVUS criteria for abnormal findings**
**IJVs**	- VH criteria 1 - presence of reflux/bidirectional flow in both sitting and supine positions	- Absent or pinpoint flow on axial TOF or TRICKS	- presence of significant stenosis (defined as venous lumen reduction ≥50 %)	- presence of significant stenosis (defined as venous lumen reduction ≥50 %)
	*and/or*	*with or without*	*with or without*	*and/or*
	- VH criteria 3 - presence of B-mode abnormalities (web, flap, membrane, malformed valve, septum) or CSA ≤0.3 cm^2^	- presence of collateral veins (external jugular veins, anterior jugular veins, facial veins, thyroid veins and deep cervical veins)	- presence of collateral veins (either epidural plexus or anterior and external jugular veins, not along the spinal cord)	*-* presence of various intra-luminal defects (webs, fibrotic annulus-like constrictions, flaps, septa, multiple channels)
	*and/or*			*and/or*
	- VH criteria 4 - absence of detectable flow			- presence of IHFD and DPL
	*and/or*			*and/or*
	- VH criteria 5- negative CSA			- reduced respiratory pulsatility
**Azygos vein/ VVs**	- VH criteria 1 - presence of reflux/bidirectional flow in both sitting and supine positions in VVs	- Absence of detectable flow in VVs	- presence of significant stenosis (defined as venous lumen reduction ≥50 %) in azygos vein	- presence of significant stenosis (defined as venous lumen reduction ≥50 %) in azygos vein
	*and/or*		*with or without*	*and/or*
	- VH criteria 4 - absence of detectable flow in VVs		- presence of collateral veins (either epidural or other collaterals)	*-* presence of various intra-luminal defects (webs, fibrotic annulus-like constrictions, flaps, septa, multiple channels)
				*and/or*
				- presence of IHFD and DPL
				*and/or*
				- reduced respiratory pulsatility

**Legend:** IJVs - internal jugular veins; VVs - vertebral veins; DS - Doppler sonography: MRV - magnetic resonance venography; CV - catheter venography; IVUS - intravascular ultrasound; VH - venous hemodynamic criteria; CSA - cross-sectional area; IHFD - intra-luminal hyperechoic filling defect; DPL - double parallel lumen.

For DS, IJV abnormal findings were positive for VH DS criteria 1, 3, 4 and 5. According to this classification, the presence of at least one of the following IJV DS anomalies were considered an abnormal IJV exam: reflux/bidirectional flow in sitting and supine positions, presence of B-mode abnormality (web, flap, membrane, malformed valve, septum), stenosis ≤0.3 cm^2^, absence of detectable flow and negative ΔCSA. Similar criteria were developed to determine VV DS abnormal findings. Positive VH criteria 1 and 4 (reflux/bidirectional flow and absence of detectable flow) were considered abnormal VV exams for DS. For MRV, IJV absent or pinpoint flow were considered abnormal findings. Abnormal flow of the VVs was classified as absent. Abnormalities of the VVs were determined only by using non-invasive testing. The azygos vein was not investigated by DS or MRV.

Abnormal findings on CV for IJVs and azygos vein were considered to have the presence of significant stenosis. IVUS IJVs and azygos vein abnormalities included the presence of stenosis, intra-luminal abnormality or reduced respiratory pulsatility.

Because there is no reliable way of non-invasively assessing the azygos vein, the VVs abnormalities detected by the non-invasive testing were compared to the azygos abnormalities detected by the invasive testing.

### Blinding, training and reading of the multimodal exams

The DS was performed by 2 trained technologists (KM and VV) with previously reported test-retest reproducibility [5,6,8]. The CV and IVUS were performed by a single interventional neurosurgeon/radiologist (AHS) who is very experienced in the diagnosis of cerebrovascular venous disease and routinely catheterizes extracranial veins in order to access intracranial veins. In phase 1 of the PREMiSe study, he optimized diagnostic workflow and resolved possible blinding issues on 10 MS subjects with the use of invasive imaging techniques (CV and IVUS) for a more accurate extracranial venous drainage assessment.

All imaging modalities were analyzed in a blinded manner by more than one viewer, upon which consensus was reached. All exams were de-identified prior to the reading so that the double-blinded readers were fully-blinded to origin and the other imaging modalities of the individual subject exams. Two independent neuroimaging experts double-read all DS and MRV examinations (KD and DH), while the CV and IVUS were double-read by two independent interpreters (AHS and YK). They had access only to the diagnostic part and not the interventional part of the study. Additional three expert neuroimaging professionals/neurosurgeons/radiologists (RZ, EL and NH) served as a panel to reach a consensus when there were discrepancies by the readers and further confirmed the correctness of the exam reading by a-priori comparing un-blinded reading results of the individual imaging modalities on a subject level.

### Statistical analyses

All analyses were carried out using IBM SPSS Statistics 20 (IBM Corp.). Demographic, clinical and hemodynamic characteristics were determined by use of frequencies.

Sensitivity, specificity, positive predictive value (PPV) and negative predictive (NPV) values were calculated using contingency tables denoting the presence or absence of vein-specific abnormality findings between all individual imaging modalities considered as the "gold standard/benchmark". We also compared combinations of noninvasive vs. invasive imaging techniques. Because the inclusion criteria for the study were the presence of ≥2 VH extracranial criteria, we were able to derive only sensitivity for comparisons between other imaging techniques and DS. The odds ratio was reported along with 95% confidence interval (CI) constructed using the normal approximation.

## Results

Table 2 shows the demographic and clinical characteristics of patients participating in the PREMiSe study. In total, 15 patients signed an informed consent in phase 1 and 30 in phase 2 after prescreening qualification procedures were completed. Of those, 5 in phase 1 and 10 in phase 2 did not fulfill noninvasive screening procedure requirements on DS. Therefore, 10 patients in phase 1 and 20 in phase 2 were enrolled in the invasive screening portion of the PREMiSe study.

**Table 2:** Demographic, clinical and Doppler sonography characteristics in multiple sclerosis patients in the PREMiSe study

	**Phase 2 (n = 20)**
Female gender, n (%)	14 (70)
Age in years, mean (SD) median	44.3 (9) 44.6
Age at onset in years, mean (SD) median	33.4 (10) 35.5
Disease duration in years, mean (SD) median	10.9 (7.1) 10
Disease course, n (%)	
RR	13 (65)
RP	7 (35)
EDSS, mean (SD) median	3.9 (1.5) 3.8
Type of treatment, n (%)	
Interferon beta	15 (75)
Glatiramer acetate	4 (20)
Combination	0 (0)
Others	1 (5)
VH CCSVI criterion, n (%)	
VH1	8 (40)
VH2	19 (95)
VH3	20 (100)
VH4	14 (70)
VH5	4 (20)
≥2 CCSVI criteria*, n (%)	20 (100)
≥2 CCSVI extracranial criteria, n (%)	20 (100)

**Legend:** PREMiSe - Prospective Randomized Endovascular therapy in Multiple Sclerosis (study); RR - relapsing-remitting; RP - relapsing-progressive; EDSS - Expanded Disability Status Scale; VH – venous hemodynamic; CCSVI -chronic cerebrospinal venous insufficiency; SD - standard deviation.

*The overall CCSVI criteria report both the extracranial and intracranial assessments on DS.

All noninvasive and invasive study procedures were well-tolerated. No intra- or postprocedural complications, including vessel rupture, thrombosis side effects to the contrast media or mortality were recorded at 24 hours or 1 month.

### Frequency of venous abnormalities on noninvasive and invasive imaging techniques in phase 2 of the PREMiSe

All 20 patients fulfilled DS screening criteria that showed anomalies in their IJVs and/or VVs (Figure 1). Of those, one patient did not fulfill invasive screening criteria for endovascular intervention (venous lumen diameter reduction ≥50%).

In particular, 19 (95%) patients showed venous abnormality of the right and 19 (95%) of the left IJV, whereas there were 7 (35%) patients who showed venous abnormality of the VVs. The MRV venous abnormalities were found in 7 (35%) of the right and 7 (35%) of the left IJVs and in 3 (15%) of the VVs (Figure 2). Seventeen (85%) patients showed the presence of collateral neck veins on MRV in the right and 15 (75%) on the left side, both on TOF and TRICKS. There were a total of 2.3 (SD 1.2, range 0–4) collateral neck veins on TOF and 2.3 (SD 1.2, range 0–4) on TRICKS.

Eleven (55%) patients showed venous abnormality on CV of the right IJV, 14 (73.6%) of the left IJV and 10 (50%) of the azygos veins (Figure 3). Of all stenotic lesions detected by CV in the right IJVs, 11 (100%) were in the lower segment (J3) and 3 (30%) were in the upper segment (J1 or J2), whereas in the left IJVs, 11 (78.6%) of the 14 stenotic veins had lesions detected in the lower segment (J3) and 10 (76.9%) in the upper segment (J1 or J2). The stenotic segment in the azygos vein was in the same location (descending part of the azygos vein distal to the azygos arch). Two of the 20 (10%) left IJVs were not examined by IVUS and one (10%) by CV because of the difficulty to access with the wire. Only 13 of the 14 cases who showed venous abnormality in the left IJV were considered for comparison between CV and IVUS, as one case overlapped with one of the 2 cases who did not get examined with IVUS. Epidural collateral veins were found in 14 (70%) right and 16 (88.9%) left IJVs and in 16 (80%) of the azygos veins. Other collaterals were less common.

In total, 10 (50%) right and 15 (83.3%) left IJVs and 17 (85%) azygos veins, demonstrated an IVUS abnormality (Figure 4). The stenosis on IVUS was detected in 7 (35%) right and 11 (61%) left IJVs and in 8 (40%) azygos veins. Reduced respiratory pulsatility was observed in 7 (35%) right and 10 (55.5%) left IJVs and in 7 (35%) azygos veins. Intraluminal abnormalities (septa, vein divided into multiple channels, IHFD and DPL) were detected in all vessels.

### Sensitivity and specificity analyses between noninvasive and invasive venous abnormality findings in phase 2 of the PREMiSe study

Table 3 shows sensitivity analyses of noninvasive and invasive imaging techniques vs. DS (as a "gold standard/benchmark") for the detection of abnormal findings in the IJVs and in azygos vein/VVs. The sensitivity of CV + IVUS was 68.4% for the right and 100% for the left IJV, compared to venous anomalies detected on DS. The sensitivity of IVUS to detect venous anomalies in azygos vein/VVs was high when compared to DS (85.7%).

**Table 3:** Comparison of Doppler sonography (as "gold standard/benchmark") for the detection of abnormal findings in the internal jugular veins and in azygos vein/vertebral veins using other noninvasive and invasive imaging techniques

**Doppler sonography**	**# of positive cases**		**Noninvasive and invasive imaging techniques**	**# of positive cases**	**Total # of cases**	**Sensitivity**
IJV right	19	20	MRV IJV right	7	20	36.8
			CV IJV right	11		57.9
			IVUS IJV right	10		52.6
			CV + IVUS IJV right	13		68.4
IJV left	19	19	MRV IJV left	7	20	36.8
			CV IJV left	14	19	73.7
			IVUS IJV left	15	18	83.3
			CV + IVUS IJV left	18	18	NA*
IJVs total	20	20	MRV IJV total	8	20	40
			CV IJV total	17		85
			IVUS IJV total	18		90
			CV + IVUS IJV total	20		NA*
VVs total	7	20	MRV VV total	3	20	28.6
			CV azygos total	10		28.6
			IVUS azygos total	17		85.7
			CV + IVUS azygos total	18		85.7

**Legend:** IJV(s) - internal jugular vein(s); VVs - vertebral veins; MRV - magnetic resonance venography; CV - catheter venography; IVUS - intravascular ultrasound.

Nineteen and18 patients successfully obtained CV and IVUS in the left IJV, respectively (because of difficulty to access with the wire). All 20 patients successfully obtained other noninvasive and invasive examinations in the explored vein territories. Therefore, for the left IJVs comparisons with CV and IVUS, only data from 19 and 18 patients were used.

*The SPSS does not provide an estimate of odds ratio or its confidence interval whenever there is a zero count in the contingency table. Instead, it returns an estimate of relative risk. Therefore those results are reported as "not available".

Table 4 shows the sensitivity, specificity, PPV, NPV and OR of noninvasive and invasive imaging techniques vs. MRV as the "gold standard/benchmark" for the detection of abnormal findings in the IJVs and in azygos vein/VVs. Compared to the venous anomalies detected on MRV, the sensitivity for the detection of venous abnormalities on CV + IVUS was 71.4% in the right and 100% in left IJVs and 100% in azygos but the specificity was 38.5%, 38.9% and 11.8%, respectively.

**Table 4:** Comparison of magnetic resonance venography (as "gold standard-benchmark") for the detection of abnormal findings in the internal jugular veins and in azygos vein/vertebral veins using other noninvasive and invasive imaging techniques

**MRV**	**# of positive cases**	**Noninvasive and invasive imaging modalities**	**# of positive cases**	**Total # of cases**	**Sensitivity**	**Specificity**	**PPV**	**NPV**	**OR**
IJV right	7	CV IJV right	11	20	35	53.8	45.5	77.8	2.92 (.41-20.89)
		IVUS IJV right	10		71.4	34.8	25	80	4 (.55-29.09)
		CV + IVUS IJV right	13		71.4	38.5	38.5	71.4	1.56 (.22-11.37)
IJV left	7	CV IJV left	14	19	57.1	23.1	28.6	50	.40 (.06-2.89)
		IVUS IJV left	15	18	100	27.3	46.7	100	1.88 (1.17-3.01)
		CV + IVUS IJV left	18	18	NA*	NA*	NA*	NA*	NA*
IJVs total	8	CV IJV total	17	20	75	8.3	35.3	33.3	.27 (.02-3.67)
		IVUS IJV total	18		100	16.7	44.4	100	1.8 (1.19-2.72)
		CV + IVUS IJV total	20		100	0	0.4	---	---
VVs total	3	CV azygos total	10	20	0	41.2	0	70	.7 (.47-1.05)
		IVUS azygos total	17		100	17.6	17.6	100	1.21 (.97-1.51)
		CV + IVUS azygos total	18		100	11.8	16.7	100	1.20 (.98-1.48)

**Legend:** MRV - magnetic resonance venography; IJVs - internal jugular veins; VVs - vertebral veins; DS - Doppler sonography: MRV - magnetic resonance venography; CV - catheter venography; IVUS - intravascular ultrasound; OR - odds ratio.

Nineteen and 18 patients successfully obtained CV and IVUS in the left IJV, respectively (because of difficulty to access with the wire). All 20 patients successfully obtained other noninvasive and invasive examinations in the explored vein territories. Therefore, for the left IJVs comparisons with CV and IVUS, only data from 19 and 18 patients were used.

*The SPSS does not provide an estimate of odds ratio or its confidence interval whenever there is a zero count in the contingency table. Instead, it returns an estimate of relative risk.

Therefore those results are reported as "not available".

The sensitivity, specificity, PPV, NPV and OR of IVUS vs. CV as the "gold standard/benchmark" for the detection of abnormal findings in the IJVs and in azygos vein/VVs is shown in Table 5. The sensitivity of IVUS ranged from 72.7% for right IJV to 90% for the azygos vein, although the IVUS showed a higher rate of venous anomalies than CV.

**Table 5:** Comparison of catheter venography (as "gold standard/benchmark") for the detection of abnormal findings in the internal jugular veins and in azygos vein/vertebral veins using intravascular ultrasound

**Catheter venography**	**# of positive cases**	**Noninvasive and invasive imaging modalities**	**# of positive cases**	**Total # of cases**	**Sensitivity**	**Specificity**	**PPV**	**NPV**	**OR**
IJV right	11	IVUS IJV right	10	20	72.7	77.8	80	70	9.33 (1.19-72.99)
IJV left	13	IVUS IJV left	15	18	84.6	20	73.3	33.3	1.38 (.10-19.64)
IJVs total	17	IVUS IJV total	18	20	88.2	0	83.3	0	1.20 (.98-1.48)
Azygos total	10	IVUS azygos total	17	20	90	20	52.9	66.7	2.25 (.17-29.77)

**Legend:** IJVs - internal jugular veins; VVs - vertebral veins; D; OR -odds ratio. Nineteen and 18 patients successfully obtained CV and IVUS in the left IJV, respectively (because of difficulty to access with the wire). All 20 patients successfully obtained other noninvasive and invasive examinations in the explored vein territories. Therefore, for the left IJVs comparisons with CV and IVUS, only data from 19 and 18 patients were used.

Table 6 shows the sensitivity, specificity, PPV, NPV and OR of invasive imaging techniques vs. DS + MRV combined as the "gold standard/benchmark" for the detection of abnormal findings in the IJVs and in azygos vein/VVs. Again, because the inclusion criteria for the study were having a presence of ≥2 VH extracranial criteria, we were able to derive only sensitivity findings. The sensitivity for the detection of venous anomalies using invasive imaging techniques did not increase compared to DS + MRV.

**Table 6:** Comparison of Doppler sonography and magnetic resonance venography (combined as the "gold standard/benchmark") for the detection of abnormal findings in the internal jugular veins and in azygos vein/vertebral veins using other invasive imaging techniques

**DS + MRV**	**# of positive cases**	**Noninvasive and invasive imaging modalities**	**# of positive cases**	**Total # of cases**	**Sensitivity**
IJV right	19	CV IJV right	11	20	57.9
		IVUS IJV right	10		52.6
		CV + IVUS IJV right	13		68.4
IJV left	20	CV IJV left	14	19	73.7
		IVUS IJV left	15	18	83.3
		CV + IVUS IJV left	18	18	NA*
IJVs total	20	CV IJV total	17	20	85
		IVUS IJV total	18		90
		CV + IVUS IJV total	20		NA*
VVs total	8	CV azygos total	10	20	25
		IVUS azygos total	17		87.5
		CV + IVUS azygos total	18		87.5

**Legend:** IJV(s) - internal jugular vein(s); VVs - vertebral veins; MRV - magnetic resonance venography; CV - catheter venography; IVUS - intravascular ultrasound.

Nineteen and18 patients successfully obtained CV and IVUS in the left IJV, respectively (because of difficulty to access with the wire). All 20 patients successfully obtained other noninvasive and invasive examinations in the explored vein territories. Therefore, for the left IJVs comparisons with CV and IVUS, only data from 19 and 18 patients were used.

*The SPSS does not provide an estimate of odds ratio or its confidence interval whenever there is a zero count in the contingency table. Instead, it returns an estimate of relative risk. Therefore those results are reported as "not available".

## Discussion

This is the first multimodal imaging study in which 2 noninvasive and 2 invasive diagnostic techniques for the detection of extracranial venous anomalies, indicative of CCSVI were applied. The main finding of the study is that invasive techniques confirmed that noninvasive DS screening was a reliable approach for identifying patients eligible for further multimodal invasive imaging testing of the IJVs. In 19 of the 20 MS patients, the extracranial venous IJV anomalies indicative of CCSVI diagnosis were confirmed on CV or IVUS. However, it has to be noted that 50% of the screened MS population did not fulfill ≥2 extracranial VH DS criteria and were therefore not eligible to undergo invasive testing, which limited the study ability to investigate specificity of DS vs. invasive imaging diagnostic techniques. Nevertheless, the findings from this multimodal study are important, as they suggests that DS can be used reliably to select those patients who may present extracranial IJV venous anomalies, indicative of CCSVI, while in the same time, it can potentially exclude those patients who should not undergo an invasive testing of the IJVs. However, the noninvasive screening methods were inadequate to depict the total amount of VV anomalies that would indirectly reflect the pathology of the azygos vein identified with invasive testing. These findings are related to the fact that we were not able to directly non-invasively assess the azygos vein. In our opinion and experience [6,8,22,23], there are no reliable, non-invasive imaging modalities at this time that would directly image the azygous vein *in vivo.* Another important finding is related to the results from the invasive portion of the study, which confirmed the existence of severe extracranial venous anomalies, indicative of CCSVI that significantly impaired blood outflow from the brain.

A growing body of evidence suggests that the majority of CCSVI pathology is confined to the intra-luminal portion of extracranial veins, which requires high-resolution DS or IVUS B-mode imaging for the visualization of these anomalies [8,26,27]. It has been shown that the presence and number of these anomalies may contribute to a higher number of collateral neck veins and functional abnormalities [8,27]. While CV is considered to be "the gold standard - benchmark" for detecting stenosis in blood vessels associated with altered blood flow, the PREMiSe study showed that CV may not be sensitive enough to reveal the exact nature of narrowed vein segments [25]. CV is a luminogram and brings little or no data regarding the vessel's intra-luminal structures because of dense opacification of the lumen with contrast, which obliterates subtle intra-luminal structures [19]. There are no consensus guidelines with respect to the use of angiographic contrast for extrcranial CV examination [28]. The recent position statement of the International Society for Neurovascular Disease on the use of angiographic contrast for the assessment of IJVs and the azygos vein on CV does not provide clear guidelines on this issue [29]. Angiographic contrast may be used diluted (1:1) or non-diluted. While the diluted contrast may allow a better visualization of endoluminal structures (valve leaflets, webs, etc.) non-diluted contrast allows a better opacification of epidural and other collaterals, as well as a better estimation of overall features of the veins [28]. In the PREMiSe study, non-diluted contrast was used. It could be that use of a diluted contrast could have produced different findings. The PREMiSe study also demonstrated the advantage of IVUS compared to CV in detecting intra-luminal abnormalities as well as the importance of including IVUS during CV examination, especially for the assessment of the azygos vein [25]. It is important to note that sensitivity of IVUS to depict extracranial venous anomalies on DS, indicative of CCSVI, was in better agreement than the CV findings, especially for the azygos vein/VVs territory. However, one of the important limitations of DS screening approach is that the azygos vein cannot be directly imaged. While the sensitivity for detecting VV anomalies on IVUS vs. DS was high, DS did not detect abnormal VV flow in 10 patients who had positive IVUS in the azygos vein. Similar limitations were observed for MRV. These results suggest that currently, available noninvasive indirect screening methods are inadequate in depicting the total amount of intra-luminal pathology of the azygos vein. The sensitivity of CV + IVUS to define total IJV pathology on DS was 100%. These findings support results from the 2 previous studies in which a higher sensitivity of DS to detect extracranial anomalies on IVUS compared to CV was found in the IJVs [27,30]. These results can also explain findings from some recent reports that found low correspondence between the DS screening assessment and the CV findings [9,31].

Zamboni et al. proposed a set of 5 VH DS criteria by which MS patients were differentiated from healthy controls with 100% specificity and sensitivity. While the original publication did not provide the exact technical procedures for the protocol application in either a research or routine clinical setting, there were recent attempts to define the standardized CCSVI DS scanning protocol [5,32,33]. These revised DS protocols propose the use of quantitative measures for the definition of functional anomalies such as blood flow velocity and volume that could be potentially more reliable in assessing the degree of venous outflow obstruction in the extracranial venous system [32,33]. They also refine originally proposed VH criteria [5,32,34] and propose the use of the central blinded DS reading [33]. In this multimodal comparison of different noninvasive and invasive imaging techniques in phase 2 of the PREMiSe study, we read findings from multimodal techniques in a blinded manner by different experts and using a panel to reach a consensus when there were discrepancies by the readers. They also confirmed the correctness of the exam reading by a-priori comparing un-blinded reading results of the individual imaging modalities on a subject level. We did not consider the assessment of the second CCSVI VH criterion (reflux in deep cerebral veins) for several reasons: 1) the reproducibility of this criterion is lower compared to the other 4 VH criteria; [6,7] 2) there is no direct anatomical extracranial correlate for performing sensitivity and specificity comparisons with other multimodal imaging techniques; 3) use of this criterion contributes to the highest variability in making a CCSVI diagnosis and 4) the direction of the blood flow in veins connecting cortical with deep veins may vary considerably as a consequence of the physiologic inter-individual variation of the cerebral venous anatomy [11]. Despite this, the results of the multimodal PREMiSe study further support the value of DS VH criteria for the screening of extracranial venous anomalies in territories of left and right IJVs.

When CV + IVUS findings were compared to MRV findings, sensitivity was high but the specificity was low, confirming our previous results [23,24]. Some other investigators used a slightly different grading system for the detection of extracranial venous anomalies on MRV and found similar sensitivity but better specificity compared to CV [35]. Therefore, the use of different MRV evaluation criteria may have yielded different sensitivity and specificity results compared to CV, IVUS and DS in the PREMiSe study. While there is still a lack of standardized guidelines for the detection of extracranial venous anomalies indicative of CCSVI on MRV, the findings from the PREMiSe study indicate that MRV should be incorporated in the armentorium of noninvasive screening techniques. Further work is needed to standardize MRV morphology criteria [6,22-24,35-37] and incorporate flow and velocity information in determining subjects at risk for the detection of extracranial venous outflow anomalies with hemodynamic consequences [38-40].

The combination of DS + MRV did not yield better reliability vs. invasive imaging techniques compared to the DS alone. However, phase 2 of the PREMiSe study included only MS patients with ≥2 VH extracranial criteria, which limited our ability to explore the additive value of MRV to DS in improving sensitivity and specificity vs. other invasive imaging techniques.

It was proposed that extracranial venous collateral circulation is a compensatory mechanism for impaired venous outflow because it bypasses blocked veins and thereby reduces resistance to drainage [6,8,41]. The PREMiSe study showed an excellent correspondence between identifying collateral veins on MRV and CV. Approximately, 70%-85% of patients presented collateral veins on the right and 75%-80% on the left side of IJV on MRV and CV respectively. In addition, 80% of patients presented with collaterals of the azygos vein on CV. These findings confirm that the presence of collaterals on MRV and CV may represent an indirect compensatory mechanism for impaired venous outflow. In the previous study, we found high specificity for distinguishing MS vs. healthy controls based on >1 of collateral veins in the neck [6].

PREMiSe was an endovascular angioplasty study that did not include healthy controls or MS patients without the presence of CCSVI diagnosis on DS. This selection bias of the included population was an important limitation of this diagnostic study, as the sensitivity and specificity findings of noninvasive vs. invasive techniques cannot be generalized to the prevalence of findings to other case–control studies. However, the main aim of this multimodal study was to define and reliably detect extracranial venous anomalies, indicative of CCSVI in the IJVs and azygos vein/VVs of patients using DS and to confirm the presence of these anomalies by using 2 invasive imaging techniques. Another potential limitation of the study is a relatively small sample size, which could skew our findings. Although PREMiSe was a limited pilot trial not powered to detect the prevalence of CCSVI in the general MS population and healthy individuals, it confirmed a general prevalence of extracranial venous anomalies, indicative of CCSVI that we have reported in large cohorts using DS and MRV [5,6,8,22].

Maybe the most important result of the PREMiSe study is that our multimodal imaging findings contradict the number of recent DS studies that reported a prevalence of CCSVI <10% in MS patients [9,11,12,16,17,33,42-44]. In fact, the invasive diagnostic portion of PREMiSe confirmed that 19 of the 20 MS patients screened as CCSVI positive by DS had severe impairment of extracranial venous outflow with significant stenosis in the IJVs and azygos veins. Future, larger, case-controlled, multicenter, multimodal, noninvasive and invasive imaging studies that will include healthy controls, MS patients and patients with other neurological diseases should determine the real prevalence of CCSVI in these cohorts.

## Conclusions

In conclusion, although the use of noninvasive methods such as DS to confirm the diagnosis of CCSVI presently remain controversial, the results from the PREMiSe study indicate that DS is a reliable approach for identifying patients eligible for further multimodal invasive imaging testing of the IJVs. However, the noninvasive screening methods were inadequate to depict the total amount of azygos vein anomalies identified with invasive testing. This pilot study of a limited sample size shows that a non-invasive and invasive multimodal imaging diagnostic approach should be recommended to depict a range of extracranial venous anomalies indicative of CCSVI. However, a lack of invasive testing on the study subjects whose results were negative on DS screening and of healthy controls, further limits generalizibility of our findings. In addition, the findings from the two invasive techniques confirmed the existence of severe extracranial venous anomalies that significantly impaired normal blood outflow from the brain.

## Competing interests

Robert Zivadinov received personal compensation from Teva Neuroscience, Biogen Idec, EMD Serono, Bayer, Genzyme-Sanofi, Novartis, General Electric, Bracco and Questcor Pharmaceuticals for speaking and consultant fees. He received financial support for research activities from Biogen Idec, Teva Neuroscience, Genzyme-Sanofi, Novartis, Bracco, Questcor Pharmaceuticals and EMD Serono.

Yuval Karmon, Karen Marr, Vesela Valnarov, Kresimir Dolic, Cheryl Kennedy, Jesper Hagemeier, and Ellen Carl have nothing to disclose.

L. Nelson Hopkins received grant/research support from St. Jude Medical and Toshiba; serves as a consultant to Abbott, Boston Scientific/Stryker, Cordis, Micrus, and W. L. Gore; holds a financial interest in AccessClosure, Augmenix, Boston Scientific/Stryker, Claret Medical Inc., Micrus, and Valor Medical; has a board/trustee/officer position with AccessClosure and Claret Medical Inc.; belongs to the Abbott Vascular speakers’ bureau; and receives honoraria from Boston Scientific/Stryker, Cleveland Clinic, Complete Conference Management, Cordis, SCAI, University of Southern California and VIVA Physicians.

Elad I. Levy received research grant support, other research support (devices), and honoraria from Boston Scientific/Stryker and research support from Codman & Shurtleff, Inc. and ev3/Covidien Vascular Therapies (6); has ownership interests in Intratech Medical Ltd. and Mynx/Access Closure; serves as a consultant on the Codman & Shurtleff board of Scientific Advisors; serves as a consultant per project and/or per hour for Codman & Shurtleff, Inc., ev3/Covidien Vascular Therapies and TheraSyn Sensors, Inc; and receives fees for carotid stent training from Abbott Vascular and ev3/Covidien Vascular Therapies. Dr. Levy receives no consulting salary arrangements. All consulting is per project and/or per hour.

Bianca Weinstock-Guttman received personal compensation for consulting, speaking and serving on a scientific advisory board for Biogen Idec, Teva Neuroscience and EMD Serono. She received financial support for research activities from NMSS, NIH (not for the present study), ITN, Teva Neuroscience, Biogen Idec, EMD Serono and Aspreva.

Adnan H. Siddiqui has received research grants from The National Institute of Health (not related to the present study) and the University at Buffalo (Research Development Award); holds financial interests in Hotspur, Intratech Medical, StimSox, and Valor Medical; serves as a consultant to Codman & Shurtleff, Inc., Concentric Medical, ev3/Covidien Vascular Therapies, GuidePoint Global Consulting, and Penumbra; belongs to the speakers’ bureaus of Codman & Shurtleff, Inc. and Genentech. He serves on an advisory board for Codman & Shurtleff; and has received honoraria from Abbott Vascular, American Association of Neurological Surgeons’ courses, an emergency medicine conference, Genentech, Neocure Group LLC.

## Authors’ contributions

Conception and design: RZ. Acquisition of Data: All authors. Analysis and Interpretation of Data: All authors. Literature Research: RZ. Drafting the Manuscript: RZ. Critically Revising the Manuscript: All authors. Final Approval of the Manuscript: All Authors. Guarantors of entire study: RZ.

## Pre-publication history

The pre-publication history for this paper can be accessed here:

http://www.biomedcentral.com/1471-2377/13/151/prepub
